# Cell Instructive Behavior of Composite Scaffolds in a Co-Culture of Human Mesenchymal Stem Cells and Peripheral Blood Mononuclear Cells

**DOI:** 10.3390/jfb15050116

**Published:** 2024-04-27

**Authors:** Georgia-Ioanna Kontogianni, Amedeo Franco Bonatti, Carmelo De Maria, Raasti Naseem, Catarina Coelho, Kalliopi Alpantaki, Aristea Batsali, Charalampos Pontikoglou, Paulo Quadros, Kenneth Dalgarno, Giovanni Vozzi, Chiara Vitale-Brovarone, Maria Chatzinikolaidou

**Affiliations:** 1 Department of Materials Science and Engineering, University of Crete, 70013 Heraklion, Greece; gi.kontogianni@gmail.com; 2 Research Center E. Piaggio, Department of Information Engineering, University of Pisa, 56126 Pisa, Italy; bonattiamedeo@gmail.com (A.F.B.); carmelo.demaria@unipi.it (C.D.M.); giovanni.vozzi@unipi.it (G.V.); 3 School of Engineering, Newcastle University, Newcastle upon Tyne NE1 7RU, UK; raasti.naseem@newcastle.ac.uk (R.N.); kenny.dalgarno@newcastle.ac.uk (K.D.); 4 FLUIDINOVA, S.A., 4475-188 Maia, Portugal; catarina.coelho@fluidinova.pt (C.C.); paulo.quadros@fluidinova.com (P.Q.); 5 Department of Orthopaedics and Trauma, Venizeleion General Hospital of Heraklion, 70013 Heraklion, Greece; apopaki@yahoo.gr; 6 Hemopoiesis Research Laboratory, School of Medicine, University of Crete, 70013 Heraklion, Greece; tea_ios@yahoo.gr (A.B.); xpontik@uoc.gr (C.P.); 7 Department of Applied Science and Technology, Politecnico di Torino, 10129 Turin, Italy; chiara.vitalebrovarone@polito.it; 8 Foundation for Research and Technology Hellas (FO.R.T.H)-IESL, 70013 Heraklion, Greece

**Keywords:** PLLA, PCL, PHBV, 3D-printed scaffolds, osteogenesis, osteoclastogenesis, bone tissue engineering, bioactive scaffolds, strontium, nanohydroxyapatite

## Abstract

The in vitro evaluation of 3D scaffolds for bone tissue engineering in mono-cultures is a common practice; however, it does not represent the native complex nature of bone tissue. Co-cultures of osteoblasts and osteoclasts, without the addition of stimulating agents for monitoring cellular cross-talk, remains a challenge. In this study, a growth factor-free co-culture of human bone marrow-derived mesenchymal stem cells (hBM-MSCs) and human peripheral blood mononuclear cells (hPBMCs) has been established and used for the evaluation of 3D-printed scaffolds for bone tissue engineering. The scaffolds were produced from PLLA/PCL/PHBV polymeric blends, with two composite materials produced through the addition of 2.5% *w*/*v* nanohydroxyapatite (nHA) or strontium-substituted nanohydroxyapatite (Sr-nHA). Cell morphology data showed that hPBMCs remained undifferentiated in co-culture, while no obvious differences were observed in the mono- and co-cultures of hBM-MSCs. A significantly increased alkaline phosphatase (ALP) activity and osteogenic gene expression was observed in co-culture on Sr-nHA-containing scaffolds. Tartrate-resistant acid phosphatase (TRAP) activity and osteoclastogenic gene expression displayed significantly suppressed levels in co-culture on Sr-nHA-containing scaffolds. Interestingly, mono-cultures of hPBMCs on Sr-nHA-containing scaffolds indicated a delay in osteoclasts formation, as evidenced from TRAP activity and gene expression, demonstrating that strontium acts as an osteoclastogenesis inhibitor. This co-culture study presents an effective 3D model to evaluate the regenerative capacity of scaffolds for bone tissue engineering, thus minimizing time-consuming and costly in vivo experiments.

## 1. Introduction

Bone is a dynamic tissue whose maintenance is regulated by the antagonistic action of bone-forming osteoblasts and bone-resorbing osteoclasts. Any metabolic abnormalities that disturb this equilibrium result in an imbalance between osteogenesis and osteoclastogenesis and may cause bone fractures [1]. Bone tissue engineering (BTE) introduced the creation of 3D scaffolds and constructs with specific structural, mechanical, and biological characteristics to favor the bone regeneration process [2]. The main polymeric categories used for the fabrication of scaffolds in BTE are natural and synthetic polymers [3]. Natural polymers provide a bioactive environment for the cells, harnessing excellent biological responses and an absence of immunogenic effects. However, due to their soft nature, they do not have the appropriate stiffness needed for BTE applications [4]. For this reason, synthetic polymers are often preferred, due to their controllable and high mechanical strength [5,6].

During the last decades, the conventional technologies for the fabrication of scaffolds for BTE applications have been replaced by additive manufacturing for the creation of 3D scaffolds layer-by-layer, controlling their macro- and micro-architecture with high fidelity [7]. Among the different additive manufacturing technologies, the most widely used is fused deposition modeling (FDM), in which a thermoplastic filament is melted into a liquid state in a liquefier head and is then selectively deposited through a nozzle to the printing bed [8]. The advantage of FDM is that it processes materials with mechanical properties close to those of native bone, with controllable architecture, providing a 3D environment that can mimic the topology of extracellular matrix (ECM) to the cells [9].

Aliphatic polyesters are a group of biocompatible and biodegradable synthetic polymers that are widely used in biomedical applications and BTE [10]. One of the most well studied polymers, used in a range of Food and Drug Administration (FDA)-approved devices, is poly l-lactide acid (PLLA) [11]. PLLA has favorable mechanical properties, with compressive strengths in the range of 2–39 MPa (close to trabecular bone, 2–12 MPa), making it suitable for load-bearing applications [12], but it has limited biofunctionality [13]. Poly(ε-caprolactone) (PCL) is a soft semi-crystalline polymer, also used in FDA-approved devices, with high ductility, widely used in BTE applications due to its biocompatibility and controllable degradation rate [14], although the lack of attachment sites produces limited cell adhesion [14]. Poly(3-hydroxybutyrate-co-3-hydroxyvalerate) (PHBV) is a hard non-toxic polyhydroxyalkanoate-type polymer naturally produced by bacteria with favorable responses in bone regeneration [15], but lacks ductility and this limits its application [16]. To this end, the blending of different polymers leads to hybrid materials that overcome the limitations of each separate material, endowing it with favorable physicochemical and mechanical properties [17].

Bioceramics have commonly been used in combination with polymeric materials to increase affinity to the host tissue [18]. Nanohydroxyapatite (nHA), the main component of native bone, is a popular choice due to its biocompatibility, osteoinductive properties, and enhanced bioactivity [19]. Moreover, the addition of nHA has been found to improve the mechanical properties of polymeric matrices [20] and neutralize the acidic by-products of the polymers, thus enhancing the bioactivity of implants [21]. The composition of nHA can be tailored in order to deliver ions, such as strontium (Sr^2+^), zinc (Zn^2+^), or magnesium (Mg^2+^), that are capable of promoting osteogenesis and enhance the anti-inflammatory and/or antibacterial properties of the materials [19]. Specifically, Sr acts through the calcium-sensing receptor and promotes the transduction of the mitogen-activated protein kinase signaling, thus improving the proliferation of osteoblasts and the formation of new bone [22]. The incorporation of Sr and the replacement of Ca in HA improves the growth kinetics of the crystal [23] and, at a concentration less than 10%, it can change the dissolution kinetics of HA, improving its biodegradability [24]. Notably, the incorporation of Sr in HA crystals not only enhances osteogenesis, but also inhibits osteoclastogenesis in vitro and in vivo, due to the enhancement of the low-density lipoprotein receptor-related protein 6 (LRP6)/β-catenin/osteoprotegerin (OPG) signaling pathway in osteoblasts. This serves as a decoy receptor for the receptor activator of nuclear factor kappa-Β ligand (RANKL) on the surface of monocytes, blocking multinucleation and osteoclast formation and action [25].

Conventionally, the in vitro evaluation of biomaterials in BTE is performed in mono-cultures of murine or human cells that have the potential to differentiate into osteoblasts, as well as precursor cells that differentiate into osteoclasts [26]. However, mono-cultures can poorly recapitulate the physiological microenvironment of bone tissue, in which osteoblasts and osteoclasts are in continuous communication to maintain bone homeostasis. Signals and interactions between various cell populations cannot be monitored in mono-cultures [27]. Therefore, the in vitro co-culture of osteoblasts and osteoclasts remains an unmet need for better physiological mimicry of their cross-talk and the mechanisms of their cooperation. Such co-cultures are valuable tools for pre-screening the bone regeneration capacity of scaffolds, prior to their in vivo validation, which is time consuming and expensive. During the last decade, co-culture systems of osteoblasts and osteoclasts have been established and used to closely mimic the bone remodeling process and to evaluate the reparative potential of biomaterials [28,29]. However, in most of them, biochemical stimulating agents were used to induce cells to differentiate into osteoblasts and osteoclasts. For the osteoblast maturation, a cocktail of l-ascorbic acid, β-glycerophosphate, and dexamethasone are required, while for osteoclasts maturation, macrophage colony-stimulating factor (M-CSF) and RANKL are needed. However, the impact of the different stimulating agents within a co-culture cannot be isolated and l-ascorbic acid, dexamethasone, and β-glycerophosphate also promote osteoclastogenesis [30,31], while M-CSF and RANKL are naturally produced by mature osteoblasts [32]. Consequently, an accurate picture of the cross-talk between the two cell populations cannot be obtained, due to the exogenous addition of cytokines into the culture media.

In this study, we fabricated 3D-printed scaffolds from PLLA/PCL/PHBV blends substituted with nHA or Sr-nHA via FDM and evaluated their osteogenic and osteoclastogenic potential in supplement-free co-cultures consisting of human bone marrow-derived mesenchymal stem cells (hBM-MSCs) and human peripheral blood mononuclear cells (hPBMCs). Mono-cultures of both cell types were used as a reference, to monitor differences compared to the co-cultures. The supplement-free culture allows for cross-talk between the two cell populations to be studied without confounding factors, providing understanding on the interactions between cells and 3D composite scaffolds containing the osteoconductive compounds nHA and Sr-nHA. Cell morphology was investigated using scanning electron microscopy and confocal laser microscopy. The differentiation potential was determined via biochemical assays, gene expression via real-time polymerase chain reaction, and immunohistochemistry was carried out for specific osteogenesis- and osteoclastogenesis-related markers.

## 2. Materials and Methods

### 2.1. Preparation of Scaffolds

PLLA (PURASORB PL38) was obtained from Corbion (Amsterdam, The Netherlands). PCL (average Mw 80,000) and PHBV (PHV content 8 mol%) were obtained from Merck (Darmstadt, Germany). Three-dimensional scaffolds from polymeric blends of PLLA/PCL/PHBV at 90/5/5%wt (designated as blend) with/without the incorporation of nHA and Sr-nHA (designated as composites) were fabricated via FDM, as previously reported [33]. Briefly, pellets of each polymer were combined at the specific ratio and their mixture was processed using a filament extruder (Microlab 10 mm twin-screw) to create a polymer filament. Two cycles of extrusion were completed to ensure solution homogeneity. Two enriched polymeric blends were also prepared with the incorporation of 2.5% *w*/*v* nHA (100% of Ca^2+^) and Sr-nHA (50% of Sr^2+^ substitution into the nHA), as previously described [34], and were designated as blend_nHA and blend_Sr-nHA. Briefly, nHA and Sr-nHA suspensions were mixed with PLLA pellets to make a paste, which was then dried out in an incubator overnight at 50 °C. Then, the coated PLLA with the inorganic phase were mixed with PCL and PHBV and the produced pellets were extruded using the same conditions as the non-enriched blend. Scaffold designation and composition are listed in Table 1.

An FDM printer was used for the 3D printing and the printed samples were cylindrical, with 5 mm diameter, 1 mm height, and an overall porosity of 40% [34]. Moreover, the material composition and the incorporated inorganic phase have been chemically characterized with FE-SEM, TGA, XRD, and EDS analysis, for the confirmation of the effective incorporation and release of Sr^2+^ in the nHA lattice, without the expected decomposition of the nHA phase due to the increased Sr^2+^ percentage [34,35].

### 2.2. Cell Culture

Human bone marrow mesenchymal stem cells (hBM-MSCs) were isolated from human bone marrow using Lymphoprep^TM^ (Axis-Shield, Oslo, Norway) density gradient medium, as previously described [36]. BM samples were collected from adults undergoing hip replacement surgery, after informed consent was provided and the institutional ethics approval was granted (license number: 26–05-2010/3910, University Hospital Heraklion Greece), and were then diluted in a 1:1 ratio in PBS. The addition of the density gradient medium through the central hole of the SepMate was followed by the addition of the diluted sample. After centrifugating at 1200× *g* for 20 min at room temperature, the hBM-MSC fraction was collected and washed twice with PBS. Finally, MSCs were purified and diluted in complete α-MEM, supplemented with 10% FBS (PAN-Biotech, Aidenbach, Germany), 2 mM L-glutamine (Gibco, Waltham, MA, USA), 100 μg/mL penicillin/streptomycin (Gibco, Waltham, MA, USA), and 2.5 μg/mL fungizone (amphotericin B) (Gibco, Waltham, MA, USA).

Human peripheral blood mononuclear cells (hPBMCs) were isolated from human whole blood (approved by the Ethics Committee of the University of Crete, protocol No. 7/29.01.2020) using the same Lymphoprep^TM^ (Axis-Shield, Oslo, Norway) density gradient medium and SepMate process, with the exception that the whole blood was initially diluted in a 1:1 ratio in PBS + 2% FBS. After centrifugation, the hPBMCs fraction was collected and washed twice with PBS containing 2% FBS. Finally, monocytes were purified and diluted in complete α-MEM, supplemented with 15% FBS (PAN-Biotech, Aidenbach, Germany), 2 mM L-glutamine (Gibco, Waltham, MA, USA), 100 μg/mL penicillin/streptomycin (Gibco, Waltham, MA, USA), and 2.5 μg/mL fungizone (amphotericin B) (Gibco, Waltham, MA, USA) and were frozen at −80 °C until use.

### 2.3. Immunophenotypic Characterization of hBM-MSCs and hPBMCs

Flow cytometry was used to immunophenotypically characterize hBM-MSCs at P2, by means of anti-CD73 (AD2; Becton Dickinson-Pharmingen, San Diego, CA, USA), anti-CD90 (F15.42; Immunotech/Coulter), anti-CD105 (SN6; Caltag, Burlingame, CA, USA), anti-CD45 (IMMU19.2; Immunotech/Coulter), anti-CD14 (RMO52; Immunotech/Coulter), and anti-CD34 (QBend10; Beckman-Coulter) monoclonal antibodies [37]. Freshly isolated hPBMCs were stained and immunophenotypically characterized with the antibodies CD45-PC7 and CD14-PE (both from Beckman-Coulter, California, CA, USA). Stained samples were then analyzed for monocytic population CD45+ and CD14+ cells. Analysis was performed in a Cytomics FC500 flow cytometer (Beckman Coulter, California, CA, USA).

### 2.4. Co-Culture of Human Bone Marrow Mesenchymal Stem Cells (hBM-MSCs) and Peripheral Blood Mononuclear Cells (hPBMCs)

Prior to cell seeding, the polymeric and composite scaffolds were sterilized in a two-step procedure, by immersion in 70% ethanol for 3 min, following UV irradiation for 30 min.

The co-culture protocol was adapted from an established protocol [38]. Briefly, the sterilized scaffolds were soaked in complete cell culture medium with the addition of 100 μM L-ascorbic acid 2-phosphate (Sigma, Darmstadt, Germany) for 45 min, before being removed and left to air dry for 1 h. Afterwards, hBM-MSCs (passage 2) were seeded on top of the scaffolds at a cell density of 2 × 10^4^ cells/scaffold and were cultured for 3 days in complete culture medium supplemented with 100 μM L-ascorbic acid 2-phosphate. After 3 days in culture, the osteogenic differentiation was induced by the addition of 10 nM dexamethasone (Sigma, Darmstadt, Germany), 10 mM β-glycerophosphate (Sigma, Darmstadt, Germany), and 50 μg/mL L-ascorbic acid 2-phosphate in the culture medium. After 14 days in culture, hPBMCs were seeded at a cell density of 5 × 10^5^ cells/scaffold and the culture medium was changed to alpha-MEM, supplemented with 7.5% FBS, 7.5% heat-inactivated human serum, 2 mM L-glutamine, 100 μg/mL pen/strep, and 2.5 μg/mL fungizone. These culture conditions have been selected to monitor the responses of both cell types in co-culture in the absence of external biochemical stimulants. Mono-cultures of both cell types served as control cultures. hBM-MSC mono-cultures were cultured under identical conditions as the co-culture, while in hPBMCs mono-cultures, 25 ng/mL M-CSF (Preprotech, Cranbury, NJ, USA) and 50 ng/mL RANKL (Preprotech, Cranbury, NJ, USA) were added to the culture medium, to guide the multinucleation and osteoclasts formation, ensuring their capacity towards this direction and for comparison with the co-culture. The co-cultures were maintained for 28 days without the addition of any supplementary growth factors, with medium changes twice weekly, and with three distinct time points—days 14/1, 28/14, and 42/28 (where the first number relates to the time of hBM-MSCs in culture and the second number indicates the time of hPBMCs in culture).

### 2.5. Cell Viability and Proliferation

#### 2.5.1. Cell Viability Assessment

The cell viability of hBM-MSCs and hPBMCs was determined using the PrestoBlue^TM^ assay (Invitrogen Life Technologies, Waltham, MA, USA), as previously described [6]; this is a resazurin-based indicator that is modified in the reducing environment of living cells and the product is a red and highly fluorescent product that can be detected photometrically. The cell viability was measured after 14/1, 28/14, and 42/28 days in culture. The percent viability was calculated as the absorbance of each type of culture (hBM-MSC mono-culture, hBM-MSCs, hPBMCs co-culture, and hPBMCs mono-culture) within each type of scaffold material (blend, blend_nHA, and blend_Sr-nHA), divided by the absorbance of each type of culture within the polymer blend (as control material) multiplied by 100.

#### 2.5.2. Adhesion and Morphology of hBM-MSCs and hPBMCs

The morphology of the hBM-MSCs and hPBMCs on the scaffolds was monitored using SEM (JEOL JSM-6390 LV, Tokyo, Japan) after 14/1, 28/14, and 42/28 days in mono- and co-cultures. At each time point, cells were rinsed twice with PBS, then fixed with 4% *v*/*v* para-formaldehyde (PFA) for 20 min at room temperature and were dehydrated in increasing concentrations (30% *v*/*v*–100% *v*/*v*) of ethanol. Cell-seeded scaffolds were then dried in a critical point drier (Baltec CPD 030, Baltec, Los Angeles, CA, USA), sputter-coated with a 20 nm thick layer of gold (Baltec SCD 050, Baltec, Los Angeles, CA, USA), and observed under a microscope at an accelerating voltage of 20 kV.

An actin distribution of fluorescently labeled hBM-MSCs and hPBMCs was observed using confocal laser microscopy. Cells were cultured in the scaffolds in mono- and co-cultures and at the corresponding time points, the medium was removed, and samples were washed with PBS. Cells were then fixed with 4% PFA for 20 min and washed with PBS. Cell permeabilization was performed with the addition of 0.1% bovine serum albumin (BSA) in 0.1% Triton-X, for 30 min. Samples were stained with tetramethyl-rhodamine B isothiocyanate-conjugated phalloidin (TRITC-phalloidin conjugate, Sigma-Aldrich, St. Louis, MO, USA) for 1 h, according to the manufacturer’s instructions. Cell nuclei were stained with 4′, 6′-diamidino-2-phenylindole dihydrochloride (DAPI, Invitrogen, ThermoFisher Scientific, Waltham, MA, USA) for 5 min. The samples were washed with PBS and observed under a confocal microscope (TCS SP8; Leica).

### 2.6. Cell Differentiation via Biochemical Analysis

#### 2.6.1. Alkaline Phosphatase (ALP) Activity

ALP activity was used as an early marker of osteogenesis after days 14/1, 28/14, and 42/28 in mono- and co-cultures of hBM-MSCs, based on a previously described protocol [39]. Briefly, at the specific time points, cells were rinsed twice with PBS and lysed with 100 μL lysis buffer (0.1% Triton-X in dH_2_O) and were subjected to two freeze–thaw cycles. An aliquot of 50 μL was mixed with 50 μL of ALP reaction solution (2 mg/mL p-nitrophenyl phosphate (pNPP, Sigma, Burlington, MA, USA) in 50 mM Tris-HCl and 2 mM MgCl_2_ at pH 10.5). Afterwards, samples were left for 1 h at 37 °C and the absorbance of the reaction was measured at 405 nm in a spectrophotometer (Synergy HTX Multi-Mode Microplate Reader, BioTek, Winooski, VT, USA). The absorbance values were translated into pNP concentrations, with the use of a calibration curve constructed from known pNP values. Normalization was performed to total cellular protein, determined using the Bradford assay (Applichem, Darmstadt, Germany) in the cell lysates.

#### 2.6.2. Tartrate-Resistant Acid Phosphatase (TRAP) Activity

Tartrate-resistant acid phosphatase (TRAP5b) activity via biochemical assay was measured in supernatants of the cultures after 14/1, 28/14, and 42/28 days in culture, as a marker of osteoclast differentiation and resorption activity [40]. Briefly, cells were rinsed twice with PBS and lysed with 100 μL lysis buffer (0.1% Triton-X in dH_2_O) and were subjected to two freeze–thaw cycles. An aliquot of 50 μL was mixed with 50 μL of TRAP reaction solution (2 mg/mL p-nitrophenyl phosphate in 0.1 M glycine with 20 mM sodium tartrate and 2 mM MgCl_2_ at pH 5). Afterwards, samples were left for 1 h at 37 °C and the absorbance of the reaction was measured at 405 nm in a spectrophotometer (Synergy HTX Multi-Mode Microplate Reader, BioTek, Winooski, VT, USA). The absorbance values were translated into pNP concentrations, with the use of a calibration curve constructed from known pNP values. Normalization was performed to total cellular protein, determined using the Bradford assay (Applichem, Darmstadt, Germany) in the cell lysates.

### 2.7. Expression of ALP and Visualization by Means of Confocal Laser Microscopy (CLSM)

The expression of ALP from hBM-MSCs on the polymeric scaffolds was used as an early marker of osteogenesis after 28/14 days in culture [41]. Briefly, cells were washed twice with pre-warmed α-MEM (basal, without supplements) for 2–3 min and were ALP stained (1/500) (ThermoFisher Scientific, Waltham, MA, USA) in basal medium. The cell nuclei of live cells were stained with Hoechst stain (ThermoFisher Scientific, Waltham, MA, USA). Samples were washed with basal medium and observed under a confocal microscope (TCS SP8; Leica).

### 2.8. Expression of TRAP and Visualization Microscopically

For the evaluation and visualization of multinucleated osteoclasts, hPBMCs in mono- and co-cultures were stained using a TRAP-staining kit (Sigma-Aldrich, Darmstadt, Germany) after day 42/28 in culture, according to the manufacturer’s instructions [42]. Cells were washed three times with PBS and were fixed with 4% PFA for 20 min and stained with the TRAP staining solution (Fast garnet base solution 0.07 mg/mL, sodium nitrite solution 0.01 M, naphthol AS-BI phosphoric acid solution 0.125 mg/mL, acetate solution 0.025 M, and tartrate solution 0.00335 M). Cells were observed using an inverted light microscope under 20× and 40× lenses (Axiovert 200, Carl Zeiss, Berlin, Germany) and the photographs were taken by employing a ProgRes CF scan camera and its compatible software ProgRes Capture Pro 2.8.8 (Jenoptik Optical Systems GmbH, Berlin, Germany).

### 2.9. Cell Differentiation via Real-Time Polymerase Chain Reaction

Total RNA isolation was performed after 14/1, 28/14, and 42/28 days of co-culture and was used for cDNA synthesis and qPCR analysis of the expression of several osteogenesis- and osteoclastogenesis-related genes. Total RNA isolation was performed using TRIZOL reagent (Invitrogen Life Technologies, Waltham, MA, USA), according to the manufacturer’s instructions. The concentration and the purity of the isolated RNA was determined using Nanodrop ND 1000 (Thermo Fisher Scientific, Waltham, MA, USA). Complementary DNA (cDNA) was synthesized with a PrimeScript RT Reagent Kit (Perfect Real Time) (TAKARA, Shiga, Japan), according to manufacturer’s instructions. The KAPA SYBR Fast Master Mix (2×) Universal (Kapa Biosystems, Merck, Darmstadt, Germany) was used for RT-PCR reactions that were performed in a CFX Connect Bio-Rad real-time PCR system (Bio-Rad, California, CA, USA), on markers of (i) bone gamma-carboxyglutamic acid-containing protein (BGLAP, osteocalcin, OSC), (ii) secreted protein acidic and rich in cysteine (SPARC, osteonectin, OSN), (iii) osteoprotegerin (OPG) for osteogenesis, and (iv) dendritic cell-specific transmembrane protein (DC-STAMP), (v) nuclear factor of activated T cells 1 (NFATC1), and (vi) tartrate-resistant acid phosphatase (TRAP) for osteoclastogenesis. The Primer-Blast software (http://www.ncbi.nlm.nih.gov/BLAST) (accessed on 10 November 2020) was used for the primer design (Table 2). Amplification profiles for PCR were optimized for primer sets. For SPARC, OPG, DC-STAMP, NFATc1, and TRAP, the real-time PCR reaction was run at 95 °C for 3 min, followed by 40 amplification cycles at 95 °C for 3 s and 58 °C for 30 s. For BGLAP, the reaction was run at 95 °C for 3 min, followed by 40 amplification cycles at 95 °C for 3 s and 56 °C for 30 s. The run was completed with the dissociation curve beginning at 65 °C for 5 s and increasing to 95 °C with 0.5 °C increments. Data were analyzed with the Bio-Rad CFX manager software version 3.0. The relative expression of target genes was calculated using the ΔΔCq (where Cq is the threshold cycle) method, after normalization to two housekeeping genes evaluated using BestKeeper (beta-2-microglobulin (B2M) and succinate dehydrogenase complex, subunit A, (SDHA)). Triplicate samples at each time point were analyzed (n = 3).

### 2.10. Statistical Analysis

Statistical analysis was performed using the one-way ANOVA Dunnett’s multi-comparison test in GraphPad Prism version 8 software (GraphPad Software, San Diego, CA, USA). *p*-values indicate statistically significant differences (* *p* < 0.05, ** *p* < 0.01, *** *p*< 0.001, **** *p* < 0.0001, ***** *p* < 0.00001).

## 3. Results

### 3.1. Scaffolds Characterization

The thermal, mechanical, and morphological features of the polymeric and composite 3D-printed scaffolds have been previous reported [34]. Briefly, the thermal properties of the scaffolds, as well as the nHA and Sr-nHA content were evaluated using thermogravimetric analysis, confirming the presence of PCL and PHBV in the polymer blend, as well as the substitution degree of nHA and Sr-nHA at 2.5% *w*/*v*. The 3D scaffolds revealed a smooth material morphology with aligned layers, affirming the accuracy of the selected printing parameters for the manufacturing.

### 3.2. Characterization of hBM-MSCs and hPBMCs

**Figure 1:**
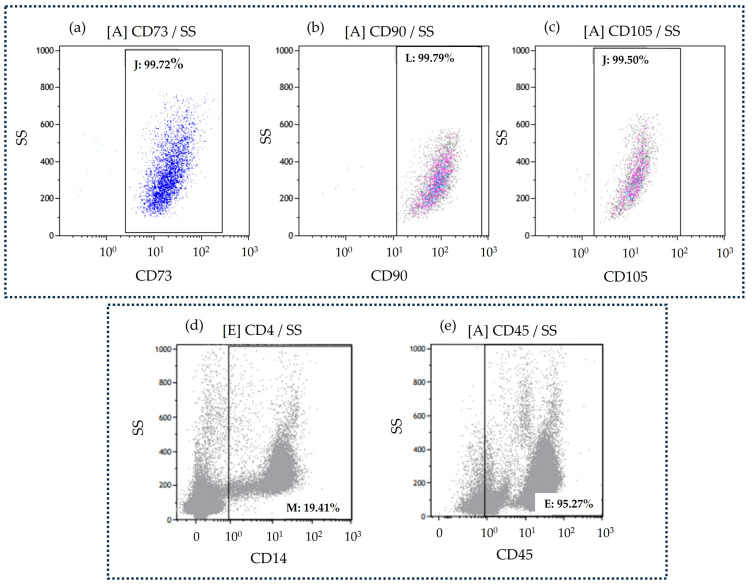
Representative flow cytometric characterization of hBM-MSCs, depicting the expression of CD73 (**a**), CD90 (**b**), and CD105 (**c**) and of hPBMCs presenting the expression of CD14 (**d**) and CD45 (**e**) (SS is side scatter).

Cultured hBM-MSCs displayed the characteristic spindle-like morphology, while the immunophenotypic analysis at passage 2 (P2) demonstrated a homogenous cell population positive for CD73 (Figure 1a), CD90 (Figure 1b), and CD105 (Figure 1c), in agreement with the established immunophenotypic profile of hBM-MSCs [43].

Isolated hPBMCs showed the characteristic round-shaped morphology and their immunophenotypic analysis demonstrated a cell population negative for CD14 (Figure 1d) and positive for CD45 (Figure 1e), as previously reported [44].

### 3.3. Cell Viability Assessment within the Scaffolds

**Figure 2:**
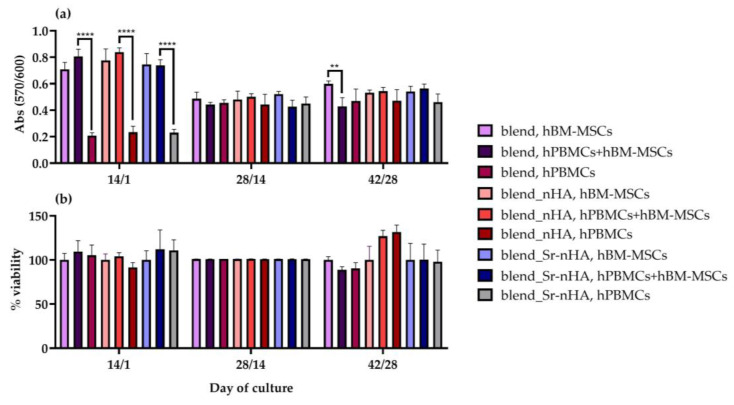
Cell viability assessment showing the (**a**) absorbance of hBM-MSCs and hPBMCs, cultured for 14/1, 28/14, and 42/28 days on polymeric and composite scaffolds and (**b**) % cell viability within the composite scaffolds, compared to the polymeric blend counterparts for the corresponding mono- and co-cultures at each time point. Each bar represents the mean ± SD of n = 4 (** *p* < 0.01, **** *p* < 0.0001).

The cell viability in mono- and co-cultures within the polymeric scaffolds was assessed quantitatively on days 14/1, 28/14, and 42/28, after cell seeding of hBM-MSCs and hPBMCs, respectively (Figure 2). At all experimental time points, the number of viable cells was similar for the polymeric blend and the composites (with nHA or Sr-nHA) (Figure 2a) for each corresponding type of culture. Additionally, for the hBM-MSC mono- and co-culture, there is a decrease from day 14/1 to 28/14 and up to day 42/28, showing culture saturation. Moreover, hPBMCs showed an increasing cell viability in the mono-cultures, due to the exogenous treatment with M-CSF and RANKL that promotes cell multinucleation and, therefore, proliferation and differentiation to multinucleated osteoclasts. The non-significant differences between hBM-MSC mono- and co-cultures indicate that hPBMCs do not affect the cell viability.

### 3.4. Cell Adhesion and Morphology

**Figure 3:**
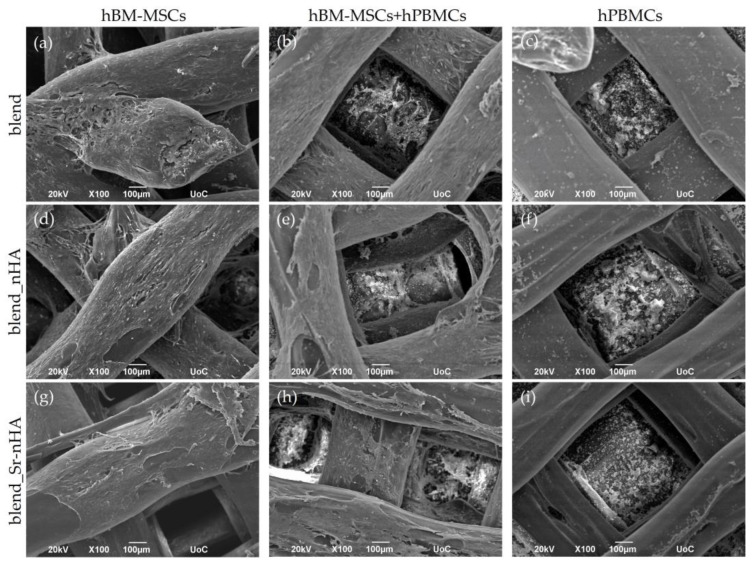
Representative SEM images showing hBM-MSCs mono-cultured on blend (**a**), blend_nHA (**d**), blend_Sr-nHA (**g**), hBM-MSCs and hPBMCs co-cultured on blend (**b**), blend_nHA (**e**), blend_Sr-nHA (**h**), and hPBMCs mono-cultured on blend (**c**), blend_nHA (**f**), blend_Sr-nHA (**i**) after 14/1 days in culture. Original magnifications are ×100 and scale bars represent 100 μm.

The cell morphology of hBM-MSCs and hPBMCs in mono- and co-cultures was investigated by means of SEM. The formation of dense layers of the osteogenically induced hBM-MSCs on the scaffold surface was noticed from day 14/1 of the co-culture in the corresponding mono- (Figure 3a,d,g) and co-cultures (Figure 3b,e,h) on blend and composite scaffolds. The presence of hPBMCs was identified as round-shaped cells in the co-culture (Figure 3b,e,h) and in the mono-culture (Figure 3c,f,i). The SEM images depict the hPBMCs at the bottom of the scaffolds, due to their limited adherence on the scaffold struts.

**Figure 4:**
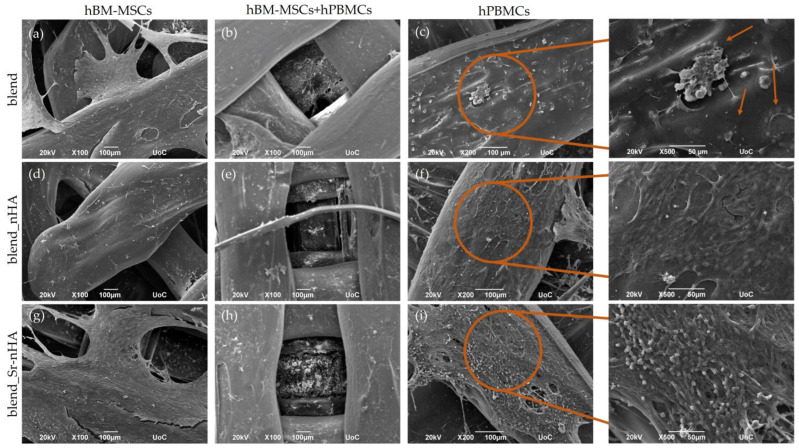
Representative SEM images showing hBM-MSCs mono-cultured on blend (**a**), blend_nHA (**d**), blend_Sr-nHA (**g**), hBM-MSCs and hPBMCs co-cultured on blend (**b**), blend_nHA (**e**), blend_Sr-nHA (**h**), and hPBMCs mono-cultured on blend (**c**), blend_nHA (**f**), blend_Sr-nHA (**i**) after 28/14 days in culture. Original magnifications are ×100 (scale bars 100 μm) and higher magnifications of hPBMCs (right column, represents the orange circles) are ×500 and scale bars represent 50 μm. Orange arrows point to multinucleated osteoclasts.

Two weeks after the cell seeding of hPBMCs, SEM images were taken for the observation of the co-culture progression and the comparison with the hBM-MSCs and hPBMCs mono-cultures (Figure 4). The images confirm the quantitative cell viability results and visualize the dense cell layer formed during hBM-MSCs’ cell proliferation (Figure 4a,b,d,e,g,h), with no significant differences from the previous time point. hPBMCs retain their viability in the co-culture (Figure 4b,e,h), without obviously formed multinucleated cells. Contrarily, in the hPBMCs mono-cultures, multinucleated osteoclasts have been formed, as expected, due to the external administration of M-CSF and RANKL and, interestingly, are more clear in the blend (Figure 4c,f,i). Higher magnification images (×500) revealed that, in the pure blend scaffolds, multinucleated cells are formed (pointed with orange arrows), while, in the composite blends, multinucleation has been initiated, although still at early stages, since mononucleated cells are also present. This is a first indication that, despite the addition of the osteoclastogenic induction media, the inorganic phase addition into composite scaffolds suppresses osteoclastogenesis.

**Figure 5:**
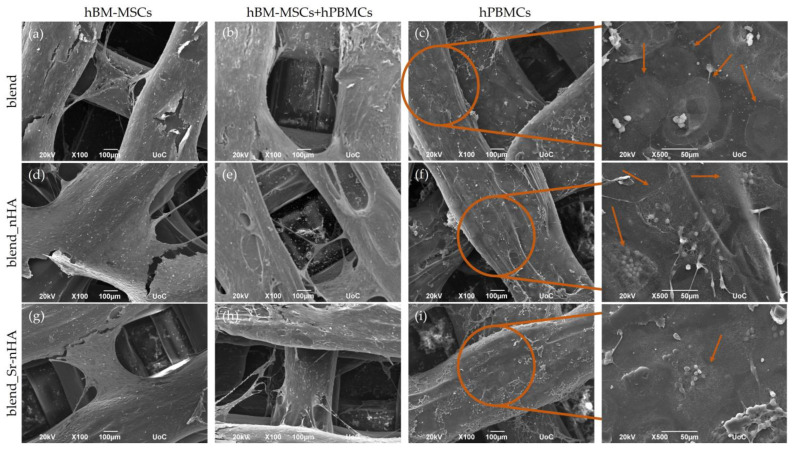
Representative SEM images showing hBM-MSCs mono-cultured on blend (**a**), blend_nHA (**d**), blend_Sr-nHA (**g**), hBM-MSCs and hPBMCs co-cultured on blend (**b**), blend_nHA (**e**), blend_Sr-nHA (**h**), and hPBMCs mono-cultured on blend (**c**), blend_nHA (**f**), blend_Sr-nHA (**i**) after 42/28 days in culture. Original magnifications are ×100 (scale bars represent 100 μm) and higher magnifications of hPBMCs (right column, magnifications of the orange circles) are ×500 (scale bars represent 50 μm). Orange arrows point to multinucleated osteoclasts.

The hBM-MSCs of both mono- (Figure 5a,d,g) and co-cultures (Figure 5b,e,h) retain their increased viability and characteristic elongated morphology on day 42/28, similarly to the time point of day 28/14, indicating high cell density on the scaffold surfaces. Interestingly, hPBMCs in the co-culture are round-shaped at the bottom of the scaffold and, thus, undifferentiated (Figure 5b,e,h). In hPBMCs mono-cultures (Figure 5c,f,i), multinucleated cells were identified, as denoted with orange arrows.

**Figure 6:**
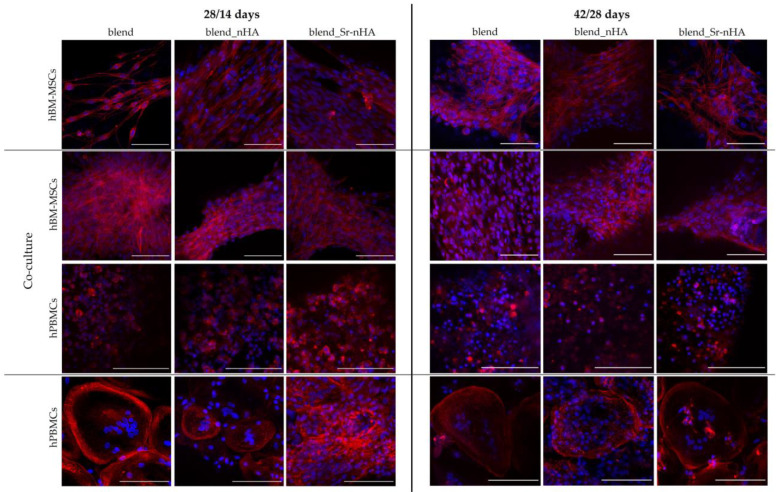
CLSM image stacks of hBM-MSCs and hPBMCs in mono- and co-cultures on blend, blend_nHA, and blend_Sr-nHA scaffolds, showing actin distribution (red) and cell nuclei (blue) after 28/14 days (left panel) and 42/28 days (right panel) in culture. Co-culture images are from two distinct locations of the scaffold, since both hBM-MSCs and hPBMCs show a preference on the scaffolds’ strands and at the bottom of the scaffold, respectively. Scale bars represent 100 μm.

The morphology of both cell types was further examined with confocal microscopy, after the staining of cell nuclei (blue) and the cytoskeletal organization via actin distribution (red) after 28/14 and 42/28 days in culture (Figure 6). These confirm that the hBM-MSCs have attached strongly on the pure and substituted polymeric surface and retain their elongated fibroblastic morphology, as expected, in both mono- and co-cultures and time points tested. The visualization of hPBMCs in the co-culture more clearly revealed the round-shaped morphology, without the formation of multinucleated osteoclasts at all polymeric materials and time points. This suggests that their direct contact with hBM-MSCs within the scaffolds and without induction media suppresses osteoclastogenesis. This observation becomes stronger in view of osteoclast formation in the hPBMCs mono-cultures in the presence of exogenous M-CSF and RANKL. Interestingly, even though formed osteoclasts were observed in the pure blend and the nHA-substituted scaffolds from day 28/14, there are no visible multinucleated osteoclasts in the Sr-nHA scaffolds at this time point, although osteoclasts have been formed in the Sr-nHA at the latest time point of day 42/28. This suggests that the Sr-nHA substitution might cause a delay in osteoclast formation, even though exogenous cytokines were added.

### 3.5. Biochemical Determination of ALP and TRAP Activity

**Figure 7:**
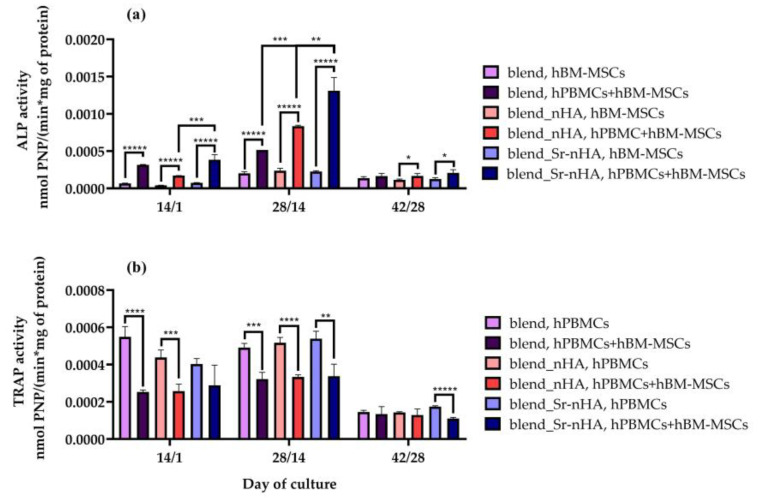
(**a**) Normalized ALP activity of hBM-MSCs and (**b**) normalized TRAP activity of hPBMCs cultured on polymeric blend, blend_nHA, and blend_Sr-nHA for 14/1, 28/14, and 42/28 days in mono- and co-cultures Each bar represents the mean ± SD of n = 4 (* *p* < 0.05, ** *p* < 0.01, *** *p* < 0.001, **** *p* < 0.0001, ***** *p* < 0.00001).

To examine the potential of hBM-MSCs cultured on the scaffold materials to differentiate into mature osteoblasts in vitro, ALP activity was measured as an early marker of osteogenesis (Figure 7a). As an early marker of osteogenesis, ALP is highly expressed at the initiation of osteoblast formation, followed by a gradual reduction after cell maturation. The ALP activity of hBM-MSC mono-cultures in all scaffold modifications was significantly lower compared to the co-cultures, at the first two experimental time points. In co-culture, the ALP activity increased until day 28/14 and decreased at day 42/28, because of the increased cell maturation. The differences between the mono- and co-cultures were higher at day 28/14 with the blend_Sr-nHA, depicting a 3-fold higher ALP activity in the co-culture compared with the corresponding mono-culture, while the enzyme activity was significantly increased when compared to the blend_nHA co-culture, indicating that the presence of Sr in the polymeric matrix enhances the osteogenic properties of the materials, as expected.

The differentiation of the hPBMCs to osteoclasts in co-culture was characterized by means of the regulation of the TRAP5b activity (Figure 7b), in order to determine the osteoclastic differentiation status in the co-culture, as well as the ability of the materials and the differentiated osteoblasts to induce multinucleation and, therefore, osteoclast differentiation in the absence of M-CSF and RANKL. Mono-cultures of hPBMCs cultured under the induction of M-CSF and RANKL were used as reference. In the co-culture, TRAP5b activity and, thus, osteoclastic differentiation of hPBMCs is significantly decreased, when compared to the corresponding mono-cultures after 14/1 and 28/14 days of culture in the presence of hBM-MSCs. Interestingly, the incorporation of Sr-nHA into the polymeric matrix kept the enzyme activity at significantly lower levels, in comparison to the mono-culture at day 42/28.

### 3.6. Expression of ALP and Visualization by Means of CLSM

**Figure 8:**
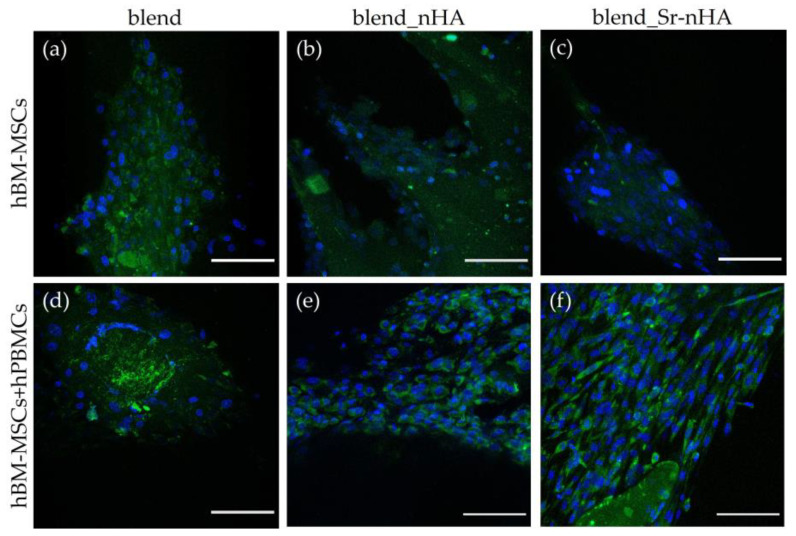
ALP live staining (green) showing hBM-MSCs in mono-culture on blend (**a**), blend_nHA (**b**), and blend_Sr-nHA (**c**), as well as in co-culture with hPBMCs on blend (**d**), blend_nHA (**e**), and blend_Sr-nHA (**f**) scaffolds after 28/14 days in culture. Cell nuclei are stained with Hoechst stain (blue). Scale bars represent 100 μm.

The osteogenic effect of the materials and the co-culture was further examined using ALP live staining at day 28/14 of culture and was visualized under CLSM (Figure 8). In accordance with the quantitative ALP activity results, live ALP staining showed increased levels in the co-culture compared to the mono-cultures, with a slight increase in the Sr-enriched scaffolds, showing a strong correlation with the addition of hPBMCs, as well as the presence of Sr.

### 3.7. TRAP Expression and Visualization

**Figure 9:**
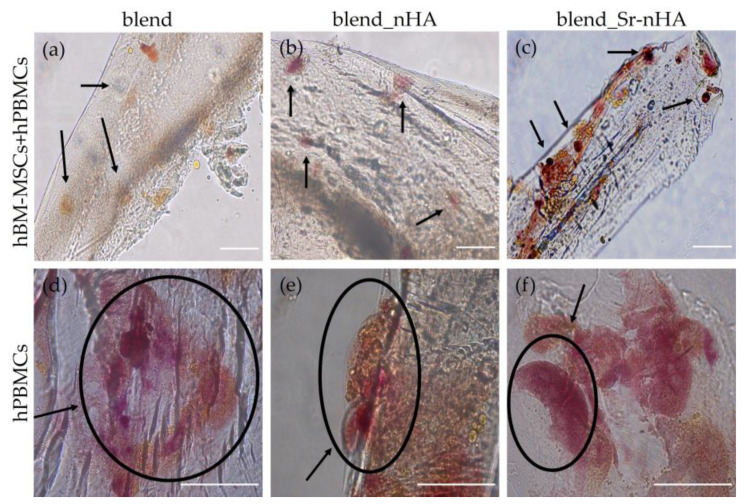
TRAP staining showing hPBMCs in co-culture with hBM-MSCs on blend scaffolds (**a**), blend_nHA (**b**), and blend_Sr-nHA (**c**) in the absence of osteoclastogenic medium and in mono-culture on blend (**d**), blend_nHA (**e**), and blend_Sr-nHA (**f**) in the presence of osteoclastogenic medium (25 ng/mL M-SCF and 50 ng/mL RANKL) after 42/28 days in culture. Black arrows show mononucleated cells, while black circles indicate multinucleated cells. Scale bars represent 100 μm.

TRAP staining in mono- and co-cultures of hPBMCs (Figure 9) revealed an absence of multinucleated osteoclasts in the co-cultures. Black arrows (Figure 9a–c) indicate characteristic round-shaped hPBMCs after 28 days of culture, corresponding to the 42/28 days of co-culture. Figure 9d–f show that hPBMCs have formed multinucleated osteoclasts in the blend and the composite scaffolds. Interestingly, there is a range of sizes of the formed osteoclasts. As previously shown, from the SEM and CLSM data, in the blend_Sr-nHA scaffolds, there is a delay in osteoclast formation, since the multinucleation process has begun without fully formed multinucleated osteoclasts, with expected sizes around 200 μm [45]. On the other hand, in blend and nHA-substituted scaffolds, osteoclasts are formed, as shown in Figure 9d,e, again indicating that strontium-substituted materials suppress osteoclast formation in mono-cultures, even in the presence of M-CSF and RANKL.

### 3.8. Analysis of Osteogenic and Osteoclastogenic Gene Expression in Culture with Pure and Substituted Polymeric Materials

**Figure 10:**
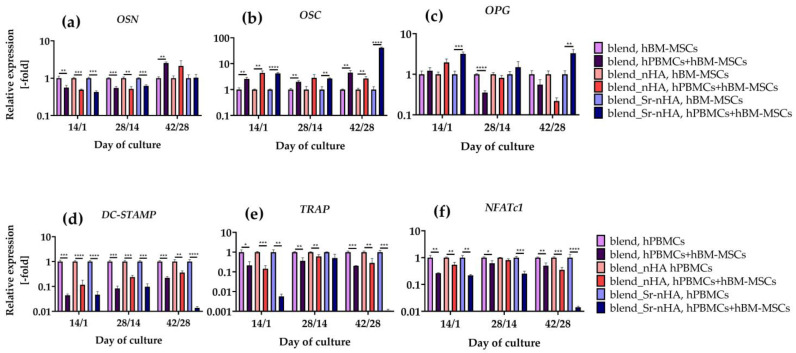
Relative osteogenic (**a**–**c**) and osteoclastogenic (**d**–**f**) gene expression of hBM-MSCs and hPBMCs, respectively, after 14/1, 28/14, and 42/28 days in mono- and co-cultures on polymeric blend, blend_nHA, and blend_Sr-nHA scaffolds. Osteonectin (OSN) (**a**), osteocalcin (OSC) (**b**), osteoprotegerin (OPG) (**c**), dendritic cell-specific transmembrane protein (DC-STAMP) (**d**), tartrate-resistant acid phosphatase (TRAP) (**e**), and nuclear factor of activated T cells 1 (NFATc1) (**f**) were analyzed. Relative gene expression levels were analyzed using the ΔΔCt method by normalizing with two housekeeping genes (B2M and SDHA) as endogenous controls. Each bar represents the mean ± SD of n = 3 (* *p* < 0.05, ** *p* < 0.01, *** *p* < 0.001, **** *p* < 0.0001).

For further investigation of the osteogenicity and osteoclastogenicity of hBM-MSCs and hPBMCs, respectively, induced by pure PLLA/PCL/PHBV 3D scaffolds or substituted with nHA or Sr-nHA, we examined the expression levels of specific osteogenic and osteoclastogenic markers. For the osteogenic differentiation of hBM-MSCs in mono- and co-cultures, the gene expression of OSN (Figure 10a), OSC (Figure 10b), and OPG (Figure 10c) was examined. For the osteoclastogenic differentiation of hPBMCs in mono- and co-cultures, the gene expression of DC-STAMP (Figure 10d), TRAP (Figure 10e), and NFATc1 (Figure 10f) was assessed.

On day 14/1 of the co-culture, OSN expression was significantly upregulated in the mono-cultures of all materials, in comparison with the corresponding co-cultures, followed by the same trend on day 28/14. On day 42/28, OSN expression was significantly higher for the pure blend in the co-culture, in comparison to the mono-culture, while, in the substituted blend with nHA and Sr-nHA, there are no significant differences between the mono- and co-cultures. The opposite trend is observed in the expression levels of OSC; on day 14/1 and day 42/28, the OSC expression was significantly higher in the co-cultures compared to the mono-cultures, while, on day 28/14, the OSC expression in blend_nHA did not show any differences between the mono- and co-cultures. The incorporation of Sr-nHA in the polymer blend caused a significant upregulation of the OPG expression in co-culture compared to the mono-culture on day 14/1 and day 42/28, while, on day 28/14, the pure blend mono-culture indicated significantly higher OPG expression levels compared to the co-culture.

All osteoclastogenesis-related markers at all tested time points and materials revealed significantly higher expression levels in the mono-cultures, in comparison to the corresponding co-cultures. Interestingly, co-cultures on Sr-nHA-substituted polymeric materials have the most significant reduction in the expression levels of DC-STAMP, TRAP and NFATc1, indicating that the presence of hBM-MSCs in combination with the presence of Sr suppresses the osteoclastogenesis more significantly and efficiently, in comparison with the other scaffold compositions.

## 4. Discussion

In this study, scaffolds from a PLLA/PCL/PHBV blend with and without the incorporation of nHA or Sr-nHA have been used for the evaluation of osteogenic and osteoclastogenic capacity in a supplement-free co-culture system in vitro. The ternary blend has been optimized and fully characterized previously [34]. Briefly, the printing parameters were optimized to produce porous scaffolds with high shape fidelity and a porosity close to the desired one, with designed pores of 800 μm. Since porosity is an important parameter in BTE applications, and open and interconnected pores are essential to provide to the cell’s nutrients and enhance waste removal, the pore size of the produced scaffolds has been shown to promote bone formation and prevent the development of fibrous tissue [46]. The pure blend scaffolds have a compressive modulus of 27 MPa, while the scaffolds substituted with nHA and Sr-nHA have 32 MPa, rendering them mechanically suitable for the regeneration of cancellous bone, which has compressive strength values of 4–12 MPa [47].

The main aim of the current study was to mimic in vitro the cross-talk and the interactions between osteoblasts and osteoclasts in a 3D environment and explore how this is affected by the polymeric blend and the composite scaffolds enriched with nHA and Sr-nHA. The co-culture period lasted for up to 28 days, with an overall time of hBM-MSC culture of 42 days. In parallel, mono-cultures of both cell types were cultured. In hPBMCs mono-cultures, M-CSF and RANKL were introduced to the cell culture medium for the guided differentiation to osteoclasts and the comparison with the co-culture populations. Previous studies have examined the interactions of bone-forming osteoblasts and bone-resorbing osteoclasts with co-culture studies that were conducted with murine osteoblasts and monocytes on silk fibroin and PLLA films [29] and hBM-MSCs and hPBMCs isolated from buffy coats on mineralized collagen [28]. In those studies, exogenous M-CSF and RANKL were added to the culture media in order to induce osteoclasts formation, affecting the natural cellular cross-talk and signaling, which could not be monitored in its original form. The cell viability assessment of the co-culture revealed that none of the examined scaffold compositions affected the cell viability and proliferation of both cell types either in co-culture or mono-culture, as evidenced by SEM and CLSM data. Similar findings have been reported in a co-culture study of hBM-MSCs and hPBMCs cultured on gelatin with HA nanospheres scaffolds [38], indicating that the presence of HA did not affect cell viability, with a mineral percentage up to 40%. SEM images at the earliest time point of day 14/1 show hPBMCs at the bottom of the scaffold in both mono- and co-cultures, which is due to the non-adherent nature of these cells [48]. As the culture period proceeds, hPBMCs in mono-cultures proliferate and multinucleate in the presence of induction factors M-CSF and RANKL, forming giant cells with clear zones and ruffles at borders, depicting a characteristic osteoclastic morphology [49]. Interestingly, in the co-cultures in the absence of induction media, hPBMCs remain round-shaped and mononucleated and, since they do not differentiate, they are visible at the bottom of the scaffold. This finding was confirmed through the CLSM images, showing a preference for hPBMCs to aggregate at the bottom of the scaffold, while hBM-MSCs aggregate at the struts. From the cell imaging data of SEM and CLSM, the absence of multinucleated osteoclasts was observed at all time points and scaffold compositions in co-culture, while, in mono-cultures, the initiation of multinucleation was visible on day 28/14. Although the hPBMCs mono-cultures were treated with osteoclastogenic medium in blend_nHA and blend_Sr-nHA scaffolds, there is a delay in osteoclast formation, indicating that the presence of the inorganic phase, and particularly Sr_nHA, suppressed cell differentiation. This behavior can be attributed to the gradual release of strontium ions from the blend_Sr-nHA scaffolds [34,35] that stimulate the calcium-sensing receptor (CaR) of hPBMCs and cause inhibition in osteoclast differentiation. Similar cell responses have been reported on PBMCs cultured in the presence of strontium ranelate with M-CSF and RANKL, showing that Sr ions activated CaR and suppressed osteoclastogenesis in a dose-dependent manner [50].

The osteogenic differentiation of hBM-MSCs was first confirmed using the ALP activity assay, which showed an increase until day 28/14 in co-culture. Osteoblasts were more active in co-culture with hPBMCs on the composite materials and especially on the scaffolds with 2.5% Sr-nHA, showing that the osteogenic differentiation is enhanced by the presence of hPBMCs and the Sr substitution. These results are in line with a previous report on PLA electro-spun scaffolds substituted with 20 and 50wt% nHA [51], displaying the effect of nHA on enhanced osteogenesis, through the expression of osteopontin, osteocalcin, and ALP. The suppression of osteoclastogenesis in co-culture was further confirmed by TRAP activity, which revealed significantly lower values in co-cultures compared to the corresponding mono-cultures at the specific time points. This observation is in line with a previous report on co-cultures of hBM-MSCs and hPBMCs on HA-substituted gelatin scaffolds [38]. The biochemical determination of these two osteogenic- and osteoclastogenic-related markers is in accordance with the ALP and TRAP staining after day 28/14 and day 42/28 in co-culture, respectively, validating that the composite materials significantly promote osteogenesis and suppress osteoclastogenesis, compared to the control polymer blend.

The effect of scaffold composition on the behavior of both cell types became more pronounced through the expression of specific osteogenesis- and osteoclastogenesis-related genes. OSC is secreted by osteoblasts and plays a pivotal role in bone mineralization and calcium homeostasis, due to the rich glutamic acid regions that have high affinity for calcium and, therefore, the nucleation of HA [52]. OSC gene expression was significantly higher in co-cultures, rather than in the mono-cultures of hBM-MSCs, in accordance with the ALP results and previous work [51], showing enhanced osteogenic differentiation due to the culture with hPBMCs and the osteoinductive properties of the scaffolds. OCN is a glycoprotein mainly secreted at the early stages of osteogenesis, with high affinity to collagen and HA [53]. Its downregulation in co-cultures is highly associated with the increased osteoblastic maturation, since its expression precedes and controls the expression of OSC [54]. OPG is a cytokine secreted by osteoblasts and inhibits osteoclastogenesis through the formation of a complex with RANKL that reduces ligand binding to the receptor activator of NF-κB (RANK), thus suppressing osteoclast formation and regulation [55]. Hence, OPG is a potent target for the treatment of bone disorders that cause bone loss, such as in osteoporosis. Interestingly, OPG expression is significantly higher in co-culture compared to the corresponding mono-culture on the Sr-nHA substituted blend, at the earliest and latest examined time points. This result strongly supports the findings that Sr incorporation into biomaterials with subsequent release causes a downregulation of osteoclast formation indirectly, through osteoblast regulation and increase in OPG in vitro and in vivo [56,57]. Notably, the osteoclastogenic gene expression was in accordance with the biochemical evaluation and the osteogenic gene expression. DC-STAMP is the key regulator of osteoclastogenesis and its knockout in mice has been shown to completely abrogate cell–cell fusion during osteogenesis [58]. NFATc1 is the master transcription regulator of osteoclast differentiation and, thus, regulates a number of osteoclast-specific genes such as TRAP [59]. Notably, all three examined osteoclastogenesis-related markers were downregulated in co-culture compared to the corresponding mono-cultures, with the lowest expression levels observed on Sr-nHA substituted scaffolds, especially on day 42/28. These results provide strong evidence that released Sr ions, in combination with the presence of hBM-MSCs, suppress osteoclastogenesis. This is in agreement with a recent study indicating that calcium/strontium phosphates with high strontium content increased osteoblast activity and inhibited osteoclast activity in vitro, through the increased expression of bone morphogenetic protein (BMP-2) and the significantly reduced RANKL/OPG ratio, respectively [60].

In summary, in this study we introduced an in vitro platform of non-stimulated, growth factor-free co-culture of hBM-MSCs and hPBMCs that has been validated for each cell type differentiation and maturation at different points. Our results showed that the incorporation of Sr in nHA and its gradual release from the blend_Sr-nHA scaffolds significantly promotes osteogenesis and suppresses osteoclastogenesis, rendering these composite 3D-printed scaffolds excellent candidates for bone tissue regeneration. Moreover, this 3D co-culture system holds promise as a valuable tool for assessing scaffolds designed for bone regeneration applications, offering a more reliable representation of the natural microenvironment of the regenerating bone tissue, minimizing time consuming and costly in vivo experiments.

## 5. Conclusions

Polymeric blend scaffolds of PLLA/PCL/PHBV (90:5:5%wt) enriched with nHA or Sr-nHA fabricated using the FDM have been utilized as a 3D microenvironment towards the evaluation of osteogenesis and osteoclastogenesis in a supplement-free co-culture model of hBM-MSCs and hPBMCs. The developed co-culture model confirmed that osteogenically differentiated hBM-MSCs were able to communicate and affect the differentiation of hPBMCs, without the external addition of RANKL and M-CSF. Both types of composite scaffolds, blend_nHA and blend_Sr-nHA, indicated an excellent biocompatibility and significantly promoted osteoblastic cell maturation, compared to the pure polymeric blend scaffolds, suppressing osteoclast formation. SEM images indicated a delayed multinucleation in both composite scaffolds in hPBMCs mono-cultures, despite the addition of induction media. Moreover, ALP activity was enhanced in both composite scaffolds in co-culture, compared to the corresponding mono-cultures and the co-culture on the pure blend. Gene expression levels showed a more pronounced osteogenic effect within the composite scaffolds and more significantly in the Sr-nHA-substituted ones, mostly after 42/28 days of the co-culture compared to the corresponding mono-culture for the OSC and OPG gene expression. Osteoclastogenesis-related markers DC-STAMP, NFATc1, and TRAP, were significantly downregulated after 42/28 days in co-culture on Sr-nHA-substituted scaffolds. Furthermore, the establishment of this co-culture system provides an ex vivo model for the in-depth examination of the cross-talk between bone cells and their signaling pathways during bone remodeling processes. This model was developed in the presence of 3D scaffolds, recapitulating the native bone microenvironment more closely. Moreover, the regenerative ability of the examined 3D-printed composite scaffolds, in combination with their superb mechanical properties demonstrate their strong potential in the treatment of bone fractures.

## Figures and Tables

**Table 1:** Scaffold designation and composition.

Scaffold Designation	Composition
blend	Polymeric blend comprising 90/5/5%wt poly(lactic acid)/poly(ε-caprolactone)/poly(3-hydroxybutyrate-co-3-hydroxyvalerate) (PLLA/PCL/PHBV)
blend_nHA	90/5/5%wt PLLA/PCL/PHBV, enriched with 2.5% *w*/*v* nHA (100% of Ca^2+^)
blend_Sr-nHA	90/5/5%wt PLLA/PCL/PHBV, enriched with 2.5% *w*/*v* Sr-nHA (50% of Sr^2+^ substitution into the nHA)

**Table 2:** Primers designed for the real-time PCR analysis of several osteogenic and osteoclastogenic differentiation-related genes and the respective amplicon size of the PCR products.

Gene Symbol	Forward (5′-3′)	Reverse (5′-3′)	Amplicon Size (bp)
B2M	TGTCTTTCAGCAAGGACTGGT	ACATGTCTCGATCCCACTTAAC	138
SDHA	GCATGCCAGGGAAGACTACA	GCCAACGTCCACATAGGACA	127
OPG	GTGTGCGAATGCAAGGAAGG	AGCAGGAGACCAAAGACACTG	209
OSC	GTGCAGCCTTTGTGTCCAAG	TCAGCCAACTCGTCACAGTC	157
OSN	GAAACCGAAGAGGAGGTGGTG	AGAAGTGGCAGGAAGAGTCGAA	196
DC-STAMP	CCACAGAGGTGTTGTCCTCC	CCACAAGGGCCCAAAAATCG	109
NFATC1	GTCTGGGAGATGGAAGCGAAAACT	CTGGTACTGGCTTCGCTTTCTCTT	111
TRAP	GGCAGGCAGGGAGGGAATAAA	AGTCACCCACGGCTACAAAGC	200

## Data Availability

The data presented in this study are openly available in ZENODO: https://doi.org/10.5281/zenodo.10060992 (assessed on 1 November 2023).

## References

[1] Berner A., Reichert J.C., Müller M.B., Zellner J., Pfeifer C., Dienstknecht T., Nerlich M., Sommerville S., Dickinson I.C., Schütz M.A. (2012). Treatment of long bone defects and non-unions: From research to clinical practice. Cell Tissue Res..

[2] Moshiri A., Oryan A. (2012). Role of tissue engineering in tendon reconstructive surgery and regenerative medicine: Current concepts, approaches and concerns. Hard Tissue.

[3] Rao S.H., Harini B.S., Shadamarshan R.P.K., Balagangadharan K., Selvamurugan N. (2017). Natural and synthetic polymers/bioceramics/bioactive compounds-mediated cell signalling in bone tissue engineering. Int. J. Biol. Macromol..

[4] Zhang Y., Wu D., Zhao X., Pakvasa M., Tucker A.B., Luo H., Qin K.H., Hu D.A., Wang E.J., Li A.J. (2020). Stem cell-friendly scaffold biomaterials: Applications for bone tissue engineering and regenerative medicine. Front. Bioeng. Biotechnol..

[5] Bharadwaz A., Jayasuriya A.C. (2020). Recent trends in the application of widely used natural and synthetic polymer nanocomposites in bone tissue regeneration. Mater. Sci. Eng. C.

[6] Georgopoulou A., Kaliva M., Vamvakaki M., Chatzinikolaidou M. (2018). Osteogenic potential of pre-osteoblastic cells on a chitosan-graft-polycaprolactone copolymer. Materials.

[7] Butscher A., Bohner M., Hofmann S., Gauckler L., Müller R. (2011). Structural and material approaches to bone tissue engineering in powder-based three-dimensional printing. Acta Biomater..

[8] Mohamed O.A., Masood S.H., Bhowmik J.L. (2015). Optimization of fused deposition modeling process parameters: A review of current research and future prospects. Adv. Manuf..

[9] Calore A.R., Sinha R., Harings J., Bernaerts K.V., Mota C., Moroni L. (2021). Additive manufacturing using melt extruded thermoplastics for tissue engineering. Methods Mol. Biol..

[10] Seyednejad H., Ghassemi A.H., van Nostrum C.F., Vermonden T., Hennink W.E. (2011). Functional aliphatic polyesters for biomedical and pharmaceutical applications. J. Control. Release Off. J. Control. Release Soc..

[11] da Silva D., Kaduri M., Poley M., Adir O., Krinsky N., Shainsky-Roitman J., Schroeder A. (2018). Biocompatibility, biodegradation and excretion of polylactic acid (pla) in medical implants and theranostic systems. Chem. Eng. J..

[12] Komal U.K., Lila M.K., Singh I. (2020). Pla/banana fiber based sustainable biocomposites: A manufacturing perspective. Compos. Part B Eng..

[13] Mazzanti V., Malagutti L., Mollica F. (2019). Fdm 3d printing of polymers containing natural fillers: A review of their mechanical properties. Polymers.

[14] Hou Y., Wang W., Bartolo P. (2022). Investigation of polycaprolactone for bone tissue engineering scaffolds: In vitro degradation and biological studies. Mater. Des..

[15] Doyle C., Tanner E.T., Bonfield W. (1991). In vitro and in vivo evaluation of polyhydroxybutyrate and of polyhydroxybutyrate reinforced with hydroxyapatite. Biomaterials.

[16] Rivera-Briso A.L., Serrano-Aroca Á. (2018). Poly(3-hydroxybutyrate-co-3-hydroxyvalerate): Enhancement strategies for advanced applications. Polymers.

[17] Hassanajili S., Karami-Pour A., Oryan A., Talaei-Khozani T. (2019). Preparation and characterization of pla/pcl/ha composite scaffolds using indirect 3d printing for bone tissue engineering. Mater. Sci. Eng. C Mater. Biol. Appl..

[18] Du X., Fu S., Zhu Y. (2018). 3d printing of ceramic-based scaffolds for bone tissue engineering: An overview. J. Mater. Chem. B.

[19] Jiang Y., Yuan Z., Huang J.J.M.T. (2020). Substituted hydroxyapatite: A recent development. Mater. Technol..

[20] Kothapalli C.R., Shaw M.T., Wei M. (2005). Biodegradable ha-pla 3-d porous scaffolds: Effect of nano-sized filler content on scaffold properties. Acta Biomater..

[21] Jaidev L.R., Chatterjee K. (2019). Surface functionalization of 3d printed polymer scaffolds to augment stem cell response. Mater. Des..

[22] Brown E.M. (2003). Is the calcium receptor a molecular target for the actions of strontium on bone?. Osteoporos. Int..

[23] Li L., Lu X., Meng Y., Weyant C.M. (2012). Comparison study of biomimetic strontium-doped calcium phosphate coatings by electrochemical deposition and air plasma spray: Morphology, composition and bioactive performance. J. Mater. Sci. Mater. Med..

[24] Christoffersen J., Christoffersen M.R., Kolthoff N., Bärenholdt O. (1997). Effects of strontium ions on growth and dissolution of hydroxyapatite and on bone mineral detection. Bone.

[25] Sun T., Li Z., Zhong X., Cai Z., Ning Z., Hou T., Xiong L., Feng Y., Leung F., Lu W.W. (2019). Strontium inhibits osteoclastogenesis by enhancing lrp6 and β-catenin-mediated opg targeted by mir-181d-5p. J. Cell Commun. Signal..

[26] Kirkpatrick C.J. (2014). Developing cellular systems in vitro to simulate regeneration. Tissue Eng. Part A.

[27] Borciani G., Montalbano G., Baldini N., Cerqueni G., Vitale-Brovarone C., Ciapetti G. (2020). Co-culture systems of osteoblasts and osteoclasts: Simulating in vitro bone remodeling in regenerative approaches. Acta Biomater..

[28] Bernhardt A., Thieme S., Domaschke H., Springer A., Rösen-Wolff A., Gelinsky M. (2010). Crosstalk of osteoblast and osteoclast precursors on mineralized collagen—Towards an in vitro model for bone remodeling. J. Biomed. Mater. Res. Part A.

[29] Jones G.L., Motta A., Marshall M.J., El Haj A.J., Cartmell S.H. (2009). Osteoblast: Osteoclast co-cultures on silk fibroin, chitosan and plla films. Biomaterials.

[30] Tsuneto M., Yamazaki H., Yoshino M., Yamada T., Hayashi S.-I. (2005). Ascorbic acid promotes osteoclastogenesis from embryonic stem cells. Biochem. Biophys. Res. Commun..

[31] Hozumi A., Osaki M., Goto H., Sakamoto K., Inokuchi S., Shindo H. (2009). Bone marrow adipocytes support dexamethasone-induced osteoclast differentiation. Biochem. Biophys. Res. Commun..

[32] Noh A.L., Yim M. (2011). Beta-glycerophosphate accelerates rankl-induced osteoclast formation in the presence of ascorbic acid. Die Pharm..

[33] Naseem R., Montalbano G., German M.J., Ferreira A.M., Gentile P., Dalgarno K. (2022). Influence of pcl and phbv on plla thermal and mechanical properties in binary and ternary polymer blends. Molecules.

[34] Kontogianni G.-I., Bonatti A.F., De Maria C., Naseem R., Melo P., Coelho C., Vozzi G., Dalgarno K., Quadros P., Vitale-Brovarone C. (2023). Promotion of in vitro osteogenic activity by melt extrusion-based plla/pcl/phbv scaffolds enriched with nano-hydroxyapatite and strontium substituted nano-hydroxyapatite. Polymers.

[35] Melo P., Naseem R., Corvaglia I., Montalbano G., Pontremoli C., Azevedo A., Quadros P., Gentile P., Ferreira A.M., Dalgarno K. (2020). Processing of sr2+ containing poly l-lactic acid-based hybrid composites for additive manufacturing of bone scaffolds. Front. Mater..

[36] Yeo C., Saunders N., Locca D., Flett A., Preston M., Brookman P., Davy B., Mathur A., Agrawal S. (2009). Ficoll-paque versus lymphoprep: A comparative study of two density gradient media for therapeutic bone marrow mononuclear cell preparations. Regen. Med..

[37] Batsali A.K., Pontikoglou C., Koutroulakis D., Pavlaki K.I., Damianaki A., Mavroudi I., Alpantaki K., Kouvidi E., Kontakis G., Papadaki H.A. (2017). Differential expression of cell cycle and wnt pathway-related genes accounts for differences in the growth and differentiation potential of wharton’s jelly and bone marrow-derived mesenchymal stem cells. Stem Cell Res. Ther..

[38] Heinemann C., Heinemann S., Rößler S., Kruppke B., Wiesmann H.P., Hanke T. (2019). Organically modified hydroxyapatite (ormohap) nanospheres stimulate the differentiation of osteoblast and osteoclast precursors: A co-culture study. Biomed. Mater..

[39] Loukelis K., Papadogianni D., Chatzinikolaidou M. (2022). Kappa-carrageenan/chitosan/gelatin scaffolds enriched with potassium chloride for bone tissue engineering. Int. J. Biol. Macromol..

[40] Gentleman E., Fredholm Y.C., Jell G., Lotfibakhshaiesh N., O’Donnell M.D., Hill R.G., Stevens M.M. (2010). The effects of strontium-substituted bioactive glasses on osteoblasts and osteoclasts in vitro. Biomaterials.

[41] Martí M., Mulero L., Pardo C., Morera C., Carrió M., Laricchia-Robbio L., Esteban C.R., Izpisua Belmonte J.C. (2013). Characterization of pluripotent stem cells. Nat. Protoc..

[42] Wei L., Ke J., Prasadam I., Miron R.J., Lin S., Xiao Y., Chang J., Wu C., Zhang Y. (2014). A comparative study of sr-incorporated mesoporous bioactive glass scaffolds for regeneration of osteopenic bone defects. Osteoporos. Int. J. Establ. Result Coop. Eur. Found. Osteoporos. Natl. Osteoporos. Found. USA.

[43] Papadogiannis F., Batsali A., Klontzas M.E., Karabela M., Georgopoulou A., Mantalaris A., Zafeiropoulos N.E., Chatzinikolaidou M., Pontikoglou C. (2020). Osteogenic differentiation of bone marrow mesenchymal stem cells on chitosan/gelatin scaffolds: Gene expression profile and mechanical analysis. Biomed. Mater..

[44] He H., Tang L., Jiang N., Zheng R., Li W., Gu Y., Wang M. (2020). Characterization of peripheral blood mononuclear cells isolated using two kinds of leukocyte filters. Transfus. Clin. Biol. J. Soc. Fr. Transfus. Sang..

[45] Tiedemann K., Le Nihouannen D., Fong J.E., Hussein O., Barralet J.E., Komarova S.V. (2017). Regulation of osteoclast growth and fusion by mtor/raptor and mtor/rictor/akt. Front. Cell Dev. Biol..

[46] Bonatti A.F.C.I., Micalizzi S., Vozzi G., De Maria C. (2021). Bioprinting for bone tissue engineering 3D Print. Orthop. Traumatol..

[47] Milazzo M., Negrini N.C., Scialla S., Marelli B., Farè S., Danti S., Buehler M. (2019). Additive manufacturing approaches for hydroxyapatite-reinforced composites. Adv. Funct. Mater..

[48] Klinder A., Markhoff J., Jonitz-Heincke A., Sterna P., Salamon A., Bader R. (2019). Comparison of different cell culture plates for the enrichment of non-adherent human mononuclear cells. Exp. Ther. Med..

[49] Salo J., Metsikkö K., Palokangas H., Lehenkari P., Väänänen H.K. (1996). Bone-resorbing osteoclasts reveal a dynamic division of basal plasma membrane into two different domains. J. Cell Sci..

[50] Caudrillier A., Hurtel-Lemaire A.-S., Wattel A., Cournarie F., Godin C., Petit L., Petit J.-P., Terwilliger E., Kamel S., Brown E.M. (2010). Strontium ranelate decreases receptor activator of nuclear factor-κb ligand-induced osteoclastic differentiation in vitro: Involvement of the calcium-sensing receptor. Mol. Pharmacol..

[51] Morelli S., Salerno S., Holopainen J., Ritala M., De Bartolo L. (2015). Osteogenic and osteoclastogenic differentiation of co-cultured cells in polylactic acid-nanohydroxyapatite fiber scaffolds. J. Biotechnol..

[52] Young M.F., Kerr J.M., Ibaraki K., Heegaard A.M., Robey P.G. (1992). Structure, expression, and regulation of the major noncollagenous matrix proteins of bone. Clin. Orthop. Relat. Res..

[53] Rosset E.M., Bradshaw A.D. (2016). Sparc/osteonectin in mineralized tissue. Matrix Biol. J. Int. Soc. Matrix Biol..

[54] Zhu Y.-S., Gu Y., Jiang C., Chen L. (2020). Osteonectin regulates the extracellular matrix mineralization of osteoblasts through p38 signaling pathway. J. Cell. Physiol..

[55] Marie P., Halbout P. (2008). opg/rankl: Role and therapeutic target in osteoporosis. Med. Sci. M/S.

[56] Atkins G., Welldon K., Halbout P., Findlay D. (2009). Strontium ranelate treatment of human primary osteoblasts promotes an osteocyte-like phenotype while eliciting an osteoprotegerin response. Osteoporos. Int..

[57] Peng S., Liu X.S., Huang S., Li Z., Pan H., Zhen W., Luk K., Guo X.E., Lu W.W. (2011). The cross-talk between osteoclasts and osteoblasts in response to strontium treatment: Involvement of osteoprotegerin. Bone.

[58] Yagi M., Miyamoto T., Sawatani Y., Iwamoto K., Hosogane N., Fujita N., Morita K., Ninomiya K., Suzuki T., Miyamoto K. (2005). Dc-stamp is essential for cell-cell fusion in osteoclasts and foreign body giant cells. J. Exp. Med..

[59] Kim J.H., Kim N. (2014). Regulation of nfatc1 in osteoclast differentiation. J. Bone Metab..

[60] Kruppke B., Ray S., Alt V., Rohnke M., Kern C., Kampschulte M., Heinemann C., Budak M., Adam J., Döhner N. (2020). Gelatin-modified calcium/strontium hydrogen phosphates stimulate bone regeneration in osteoblast/osteoclast co-culture and in osteoporotic rat femur defects—In vitro to in vivo translation. Molecules.

